# Resurgence of influenza A after SARS-CoV-2 omicron wave and comparative analysis of hospitalized children with COVID-19 and influenza A virus infection

**DOI:** 10.3389/fmed.2023.1289487

**Published:** 2024-01-11

**Authors:** Fen Lin, Man-Tong Chen, Lin Zhang, Min Wu, He Xie, Ze-Xiang Guan, Zhe Yang, Zhong-Xian Cai, Jin-Zhou Wen, Li-Ye Yang

**Affiliations:** ^1^Precision Medical Lab Center, Chaozhou Central Hospital Affiliated to Southern Medical University, Chaozhou, Guangdong, China; ^2^Department of Pediatrics, People’s Hospital of Yangjiang, Yangjiang, Guangdong, China; ^3^Department of Pediatrics, Chaozhou Central Hospital Affiliated to Southern Medical University, Chaozhou, Guangdong, China; ^4^Chaozhou Center for Disease Control and Prevention, Chaozhou, Guangdong, China; ^5^Precision Medical Lab Center, People’s Hospital of Yangjiang, Yangjiang, Guangdong, China

**Keywords:** influenza A (Flu A), SARS-CoV-2 omicron, pediatric patients, clinical feature, laboratory indexes, benign acute childhood myositis (BACM)

## Abstract

**Context:**

The highly infectious Omicron variant of severe acute respiratory syndrome coronavirus 2 (SARS-CoV-2) have caused large-scale transmission from Dec 2022 to Feb 2023 in China. After this event, a remarkable surge of influenza A (Flu A) occurred from March to May 2023, especially in pediatric patients.

**Objectives:**

This study aimed to investigate the differences between pediatric patients infected with COVID-19 Omicron and Flu A virus.

**Methods:**

A total of 1,063 hospitalized children who admitted into two tertiary general hospital of Guangdong province of China were included. Medical records were compared retrospectively in these patients during the pandemic periods of SARS-CoV-2 omicron and Flu A.

**Results:**

A total of 592 Patients with Flu A were mostly preschool and school-aged (>3y, 76.0%), they showed higher ratio of high fever (≥39°C), cough, rhinorrhea, and vomiting than patients with SARS-CoV-2 omicron. Most of the 471 Omicron patients were young children (0–3y, 74.5%) and had more poor appetite and dyspnea symptom. Benign acute children myositis (BACM) was only observed in patients with Flu A, and a significant male predominance. Multisystem inflammatory syndrome in children (MIS-C) was only found in patients with SARS-CoV-2 Omicron. Compared to the SARS-CoV-2 Omicron group, for both age groups (0–3 years and > 3 years), the Flu A group showed significantly reduced lymphocyte (Lym) counts (*P* < 0.001), and elevated levels of aspartate aminotransferase (AST), lactate dehydrogenase (LDH), and creatinine kinase-MB (CK-MB) in laboratory indexes (all *P* < 0.001). Additionally, it was found that more children hospitalized with COVID-19 had increased C-reactive protein (CRP) levels compared to those with Flu A.

**Conclusion:**

Influenza A infections have notably surged in children, coinciding with the relaxation of COVID-19 related social restrictions. During the epidemic periods of Omicron and Flu A virus infection, different clinical and laboratory characteristics were observed in hospitalized children.

## Introduction

Flu viruses and SARS-COV-2 were demonstrated which ease of person-to-person transmission through respiratory droplet route. The clinical presentations caused by these two viruses are similar, including symptoms that range from mild forms, such as fever, cough, and in severe cases, can lead to pneumonia (1, 2). However, the pathogenesis and receptors of these two viruses are different (3, 4).

The implementation of restrictions and non-pharmaceutical measures on international travel to mitigate the spread of coronavirus disease 2019 (COVID-19), which have impacted the spread and seasonality of several respiratory viruses worldwide, including influenza A virus (5–7). According to the data on Flu infections and deaths in mainland China which reported by the Bureau of Disease Prevention and Control of the National Health and Health Commission, the influenza epidemics were concentrated mainly in the winter season (Jan–Mar and Nov–Dec) in former years (8). From Dec 2020 to Dec 2022, prevention and control measures for COVID-19 also effectively suppressed the spread of other respiratory viruses, such as flu to some extent. At the same time, due to the fact most people consciously stay at home, work at staggered hours or stop working voluntarily after being infected with SARS-CoV-2, and most primary and secondary schools and childcare institutions were closed, the risk of population gathering and virus transmission was reduced, therefore the rate of flu infections decreased in 2020 and 2021, and no usual peak was observed in the winter (9). However, with the gradually relaxed policies on prevention and control of COVID-19 infection at early 2023 in China, population flow and social activities became normalized, flu activity which mainly of the H1N1 subtype rapidly increased at the end of Feb 2023, the influenza epidemic peaked in March across many Chinese places (10). During this period, many children were infected and some schools were forced to close due to the flu.

To date, studies on comparing pediatric patients who were diagnosed with Flu A and COVID-19 are limited in China. Guangdong is one of the most populous and economically advanced metropolises in China, with a population of nearly 125 million. We utilized experiences from the epidemic of the region to provide additional guidance for the early diagnosis, treatment, and prevention of Flu A and COVID-19 infection. Here, we conducted a cross sectional study comparing the clinical and laboratory features of these two viral infections in hospitalized children at two comprehensive hospitals in Guangdong China.

## Materials and methods

### Study population

A total of 1,063 hospitalized children admitted to two tertiary general hospitals in Guangdong, China (Chaozhou Central Hospital and People’s Hospital of Yangjiang) were included in this study. Among them, 592 children (269 cases in Chaozhou and 323 cases in Yangjiang) were diagnosed with Flu A between Mar 1, 2023, and Apr 30, 2023. Additionally, 471 children (286 cases in Chaozhou and 185 cases in Yangjiang) were diagnosed with SARS-CoV-2 Omicron infection between Dec 19, 2022, and Feb 1, 2023. The diagnostic criteria for children with COVID-19 was based on the updated diagnosis and treatment guidance for SARS-CoV-2 infection (Trial Version 10) issued by the China National Health Commission (11).

This study followed the ethical guidelines of the Declaration of Helsinki and approved by the Institutional Review Board of Chaozhou Central Hospital (No. 2023009) and Yangjiang People’s Hospital (No. 20230003). Signed informed consent of participants or their guardians was waived because of the nature of retrospective study.

### Clinical data collection and definitions

In this study, electronic medical records for all patients were reviewed. Data collected included demographic details, medical history, underlying comorbidities, symptoms and signs, laboratory findings, radiology examinations, treatment measures, requirement for ventilator support, and occurrence of adverse events.

The criteria for children with Flu A or COVID-19 were: (1) age < 18 years old; (2) positive for SARS-CoV-2 nucleic acid in nasal swab and/or oropharynx swab; diagnosed with Flu A by positive nucleic acid detection and/or antibody immunofluorescence assay results; (3) relevant clinical manifestations and imaging presentations. The date of disease onset was defined as the day when the symptom was noticed. Further, pneumonia was defined as the presence of symptoms or signs including cough, abnormal lung auscultation findings and pulmonary infiltrates on chest imaging. Diagnostic criteria for benign acute childhood myositis was children presenting with sudden gait-related abnormalities or refusal to bear weight after a viral illness, neurologic findings are usually normal and creatinine kinase level is elevated, with conservative treatment measures, the condition usually resolves spontaneously within a week and without residual sequelae (12). Patient files were reviewed to identify cases of multisystem inflammatory syndrome in children (MIS-C) based on relevant literature references (13, 14).

Since our focus was on patients infected with either the single SARS-CoV-2 virus or the influenza A virus, data on co-infection with both of these viruses were excluded from this study.

### Methods

Clinical and laboratory results were analyzed and compared between hospitalized children for SARS-CoV-2 Omicron and Flu A infection. Considering that the complete blood count in children differ with increasing age, the study patients were classified into two groups for comparative analysis of laboratory results. Patient 0–3 years old were classified as group 1, patient > 3 years as group 2.

All patients in this study were tested for SARS-CoV-2 at admission. In brief, RNA was extracted from the collected swab samples. RT-PCR assay was performed for SARS-CoV-2 nucleic acid using a fluorescence-based quantitative PCR kit in two hospitals. Six common respiratory viruses (Flu virus A and B, parainfluenza virus, respiratory syncytial virus, rhinovirus and adenovirus) were tested in those suspected cases by PCR assay in Chaozhou Central Hospital, and immunofluorescence assay for seven common respiratory viruses (Flu virus A and B, parainfluenza 1,2,3, respiratory syncytial virus, and adenovirus) were performed in People’s Hospital of Yangjiang.

### Statistical analysis

All statistical analyses were performed using SPSS 20.0 software. Categorical variables were described as frequency and percentages, and continuous variables were described as mean ± SE. Differences in continuous variables between groups were analyzed with independent group *t*-tests when the data were normally distributed; otherwise, the Mann-Whitney test was utilized. Differences in categorical variables between two groups were compared by Chi-square test or Fisher’s exact test as appropriate, and *P* < 0.05 was considered statistically significant.

## Results

### Clinical features

We analyzed a total of 471 children with COVID-19 infection and 592 cases with Flu A infection, ranging in age from 1 month to 13 years old. As it was not standard practice to test for particular SARS-CoV-2 variants in all patients, we relied on aggregate national data from the Guangdong Center for Disease Control (CDC) to conduct our analysis. Based on this information, the predominant strains circulating in the pandemic are SARS-CoV-2 Omicron (15).

**FIGURE 1:**
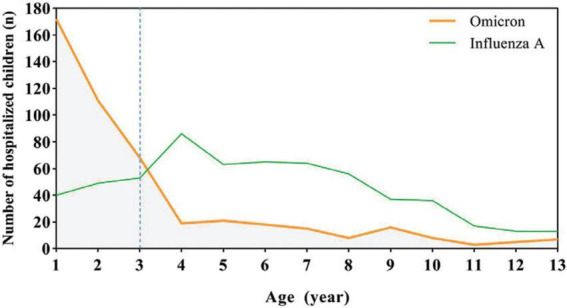
Age distribution of influenza A and SARS-CoV-2 Omicron cases in the pediatric department.

As shown in Table 1 and Figure 1, in pediatric patients with Flu A were mostly preschool and school-aged (76.0%), the majority of COVID-19 were under 3 years old (74.5%). We found no statistically significant differences in sex ratio, length of hospital stays and use of mechanical ventilator support between Flu A or COVID-19 patients. Flu A patients had higher ratio of high fever (≥39°C), cough, rhinorrhea and vomiting than SARS-CoV-2 Omicron patients (*P* < 0.01). But SARS-CoV-2 Omicron patients had more poor appetite and dyspnea symptom. Acute benign myositis was only observed in children with Flu A, and a significant male predominance, while MIS-C was only found in SARS-CoV-2 Omicron patients.

**TABLE 1:** Comparison of demographics and clinical features between hospitalized children with Omicron variant and influenza A infection.

Characteristics	Omicron (*n* = 471)	Influenza A (*n* = 592)	*P-*value
Age on admission			<0.001
0∼3y	351 (74.5)	142 (24.0)	
> 3y	120 (25.5)	450 (76.0)	
Gender			0.111
Male	288 (61.1)	390 (65.9)	
Female	183 (38.9)	202 (34.1)	
Length of hospital stay (day, mean ± SE)*	4.22 ± 0.12	4.08 ± 0.07	0.290
**Clinical symptoms**			
Fever	428 (90.9)	574 (96.9)	<0.001
peak temperature ≥ 39°C	299 (69.9)	493 (83.3)	<0.001
Fever ≥ 5d	65 (15.2)	103 (17.4)	0.110
**Respiratory symptoms**			
Cough	348 (73.9)	533 (90.0)	0.012
Sore throat	54 (11.5)	92 (15.5)	0.055
Nasal stuffiness	86 (18.3)	138 (23.3)	0.045
Rhinorrhea	126 (26.8)	213 (36.0)	0.001
Dyspnea	69 (14.6)	41 (6.9)	<0.001
**Gastrointestinal symptoms**			
Vomiting	68 (14.4)	162 (27.4)	<0.001
Diarrhea	39 (8.3)	39 (6.6)	0.293
Poor appetite	240 (51)	250 (42.2)	0.005
Pneumonia (chest CT imaging)	159/352 (45.2)	84/306 (27.5)	<0.001
Bilateral patchy shadowing	64/159 (40.3)	18/84 (21.4)	0.003
Unilateral patchy shadowing	95/159 (59.7)	66/84 (78.6)	0.003
**Clinical diagnosis**			
Febrile seizure	141 (29.9)	141 (23.8)	0.025
Acute laryngitis	214 (45.4)	116 (19.6)	<0.001
Benign acute childhood myositis	0 (0.0)	21 (3.5)	<0.001
MIS-C^▲^	12 (2.5)	0 (0.0)	<0.001
Myocarditis	9 (1.9)	14 (2.4)	0.613
Liver function damage	13 (2.8)	7 (1.2)	0.060
Previous comorbidities^†^	13 (2.8)	9 (1.5)	0.158
**Co-infection**			
Other respiratory viruses ^△^	5/113 (4.4)	7/471 (1.5)	0.062
Oxygen therapy			>0.999
Nasal catheter oxygen inhalation	97 (20.6)	125 (21.1)	
Non-invasive mechanical ventilation	3 (0.6)	4 (0.7)	
Outcome			0.587
Cure/discharge	469 (99.6)	591 (99.8)	
Death (in-hospital mortality)	2 (0.4)	1 (0.2)	

Data are n (%).

*Excluding nine COVID-19 patients and six patients with influenza A who had not cured and discharged from the hospital.

^▲^MIS-C: multisystem inflammatory syndrome in children.

^†^Omicron group: includes epilepsy (three cases), type 1 diabetes (two cases), congenital heart disease (two cases), hyperthyroidism (one case), autoimmune anemia (one case), cerebral palsy (one case), gastrointestinal bleeding (one case), adrenocortical hypofunction (one case) and autoimmune encephalitis (one case). Influenza A group: includes epilepsy (six cases), cerebral palsy (one case) and nephrotic syndrome (two case).

^△^Omicron group: includes parainfluenza virus (two cases), respiratory syncytial virus (one case), rhinovirus (two cases) and adenovirus (one case). Influenza A group: includes respiratory syncytial virus (one case), rhinovirus (four cases) and adenovirus (two case).

The epidemiologic and clinical features between Flu A and SARS-CoV-2 Omicron in hospitalized children were compared, similarities and differences were presented in Table 1.

### Laboratory and imaging findings

Regarding the imaging characteristics, significantly difference of CT scan presentation was observed among the SARS-CoV-2 omicron and Flu A patients. Specifically, 40.3% (64/159) confirmed COVID-19 infection showed bilateral pulmonary lesions, in contrast to 21.4% (18/84) clinical diagnosed Flu A patients (Table 2). Furthermore, Flu A ground-glass opacity has a central, peripheral, or random distribution. In contrast, COVID-19 ground-glass opacities have frequently been placed in the periphery of lower lobes.

**TABLE 2:** Radiologic findings of hospitalized children with COVID-19 and Influenza A.

	Confirmed cases			
	All patients (*n* = 1,063)	SARS-CoV-2 Omicron (*n* = 471)	Influenza A (*n* = 592)	*P*-value
**Radiologic finding**				
Abnormalities on chest CT No. /total No. (%)	243/658 (36.9%)	159/352 (45.2%)	84/306 (27.5%)	<0.001
Scattered inflammation shadowing	212/243 (87.2%)	133/159 (83.6%)	79/84 (94.0%)	0.021
Lung consolidation	31/243 (12.8%)	26/159 (16.4%)	5/84 (6.0%)	

**FIGURE 2:**
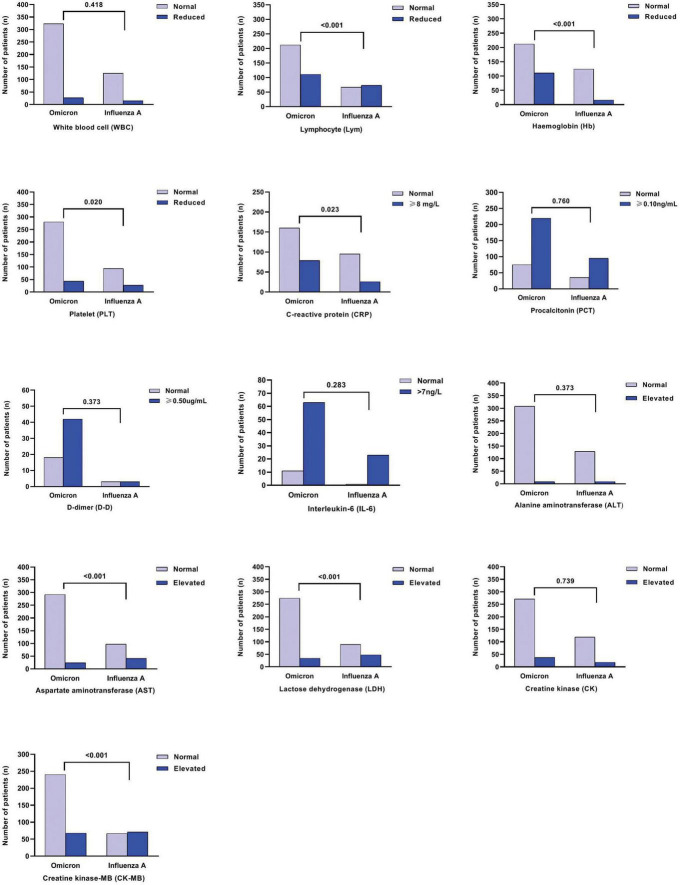
Comparison of laboratory indices among influenza A and SARS-CoV-2 Omicron groups for 0–3y old children.

**FIGURE 3:**
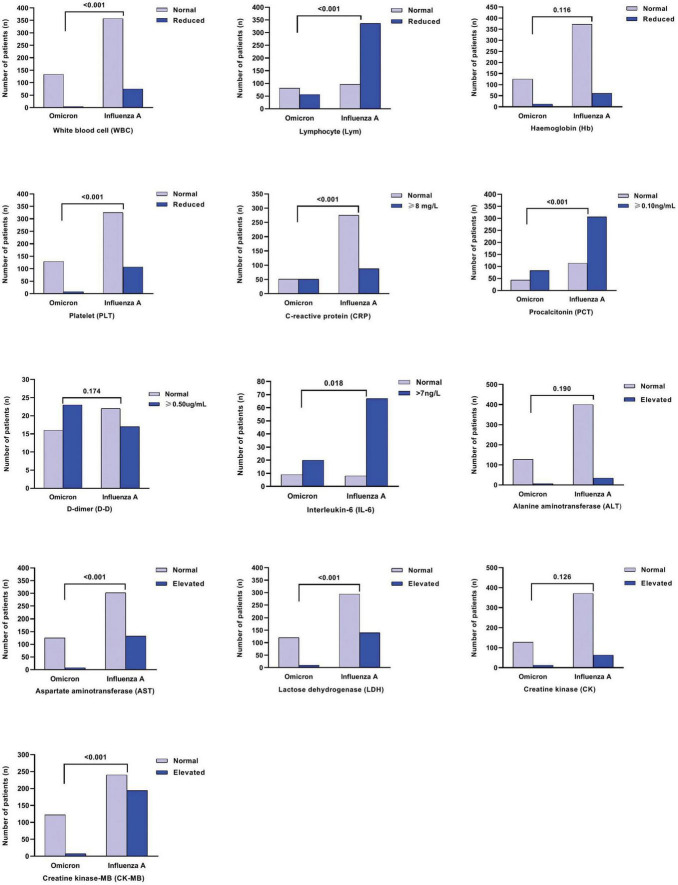
Comparison of laboratory indices among influenza A and SARS-CoV-2 Omicron groups for over 3y old patients.

As depicted in Figures 1, 2, for both age groups (0–3 years and > 3 years), the Flu A group exhibited significant lower lymphocyte (Lym) counts (*P* < 0.001), as well as higher levels of aspartate aminotransferase (AST), lactate dehydrogenase (LDH), and creatinine kinase-MB (CK-MB) (all *P* < 0.001), in comparison to the SARS-CoV-2 Omicron group. These differences in laboratory indices were statistically significant (*P* < 0.05). However, it was found that more children hospitalized with COVID-19 had increased C-reactive protein (CRP) levels compared to those with Flu A (*P* < 0.05) (Figure 3).

Additionally, other respiratory virus co-infection was confirmed by PCR or immunofluorescence assay in 4.4% omicron (5/113) cases and 1.5% (7/471) in Flu A patients (*P* = 0.062), which were parainfluenza virus, rhinovirus, and respiratory syncytial virus, respectively.

### MIS-C and BACM

During hospitalization, 12 severe/critical patients with COVID-19 developed multisystem inflammatory syndrome (MIS), which was characterized by elevated inflammatory markers such as CRP, IL-6, and PCT, and the majority of the cases had lymphopenia. All patients had fever, and four required mechanical ventilation. Other complication included convulsions, hepatic injury, respiratory failure, heart failure, myocardial injury, diffuse intravascular coagulation, and septic shock. Unfortunately, two patients deteriorated and ultimately succumbed to the illness.

Out of 63 cases of elevated CK values, 21 cases corresponded to BACM-compatible clinical presentations. There was a significant male predominance (19/21, 90.5%) with a median age of 7 years. These patients were treated conservatively, and all of them recovered without any complications.

## Discussion

In the past 3 years, China has taken relevant prevention and control measures in response to the COVID-19 pandemic, which also controlled the influenza epidemic in a low state simultaneously. An analysis of influenza and universal vaccine research in China indicated that the number of seasonal influenza infections and associated deaths after 2019 were lower than before the emergence of SARS-CoV-2 (8). A previous study involving 6,201 children from the Chaoshan area who were hospitalized with respiratory tract infections reported the lower number of influenza cases in 2020 and 2021 (Flu A detection rate of 1.9% in 2020 and 0% in 2021) (16). The public’s overall awareness of influenza prevention and control has generally declined, leading to a low rate of influenza vaccine uptake. Consequently, the population’s immunity against the influenza virus has decreased. In early 2023, China began gradually easing the stringent prevention and control measures implemented for COVID-19 infections. However, with increased movement of individuals, there was a rapid spread of the Flu A epidemic among the population. As compared to other countries, China bears a significant burden of influenza morbidity and mortality due to its large population size. Previous studies have highlighted that children play a crucial role as “drivers” of Flu epidemic (17). Therefore, it is important to identify the distinguishing features of children infected with Flu A and SARS-CoV-2, as it will aid in providing appropriate patient care and facilitating an effective public health response.

Overall, this cohorts of children infected with COVID-19 were mainly 0–3 years old. Conversely, Flu A virus affects children were mostly age over 3 years old. Evidence from three influenza epidemics (Hong Kong Flu, Asian Flu, and Spanish Flu) has consistently shown that school-age children exhibited the highest infection rates, and they also served as the primary source of adult infections (18). School-age children learn and play in classrooms, the environment is highly clustered, which is prone to transmission. Influenza vaccine of our country is a second-class vaccine with a low voluntary vaccination rate. Based on vaccination registration data in 2021 and 2022, the influenza vaccination rates of children aged 0–3 in Chaozhou area were 22.55 and 35.76%, respectively, while children aged 3–18 were only 1.23 and 3.22%, respectively (19). To promote influenza vaccination, it is recommended to gradually expand the scope of vaccine recipients and enhance public awareness. Encouraging children to actively receive the vaccine is essential, and it is advisable for schools to check the vaccination status of students at the beginning of the autumn semester admission.

Our present study revealed that fever, cough and poor appetite were common symptoms in Flu A, which is similar with COVID-19. But Flu A patients were more likely to be fever with higher temperature (≥39°C). Meanwhile, dyspnea was more likely to experience in patients with COVID-19. Lymphopenia was the common laboratory abnormality in COVID-19 adult, and it was associated with increased disease severity of COVID-19 (20). In our study, lymphocyte count was significant lower in influenza A than in COVID-19 patients, which could prove that current SARS-CoV-2 omicron infection is milder than influenza A. Additionally, we found that bilateral pulmonary lesion was more common in COVID-19 patients than in Flu A patients. Careful Chest CT illustrates dissimilarity in between, Flu A ground-glass opacity has a central, peripheral, or random distribution. In contrast, COVID-19 ground-glass opacities have frequently been placed in the periphery of lower lobes. Therefore, these few differential pathological changes may contribute distinguish imaging characteristics between Flu A and COVID-19.

Benign acute children myositis regularly occur during influenza season, characterized by a self-limited sudden onset of calf pain which causes difficulty walking. It mainly affects pre-school and school-aged boys at a median age of 6–9 years (21). During the hospitalization, we noted that 21 patients (19 males) with Flu A experienced BACM. The characteristics such as male predominance and median age (7 years old) are similar to the published reports. The cause of male predominance is unclear, but it could be related to genetic predisposition of male gender or a greater physical activity.

In the present study, BACM patients had an increased CK level (median of 1202 IU/L), mostly consistent with median values reported in some previous studies (22–24). During and after the outbreak of SARS-CoV-2, the seasonality of virus infections has changed. Clinicians should take into consideration CK levels, and BACM as a potential diagnosis when encountering children with a recent history of Flu and main complaints of calf pain during inpatient admission. All patients recovered fully, emphasizing the fact that BACM is benign, self-limited, and with an excellent prognosis.

Multisystem inflammatory syndrome in children is a rare post-infectious hyperinflammatory disorder associated with SARS-CoV-2 (25). Despite limited reports of MIS-C during the SARS-CoV-2 Omicron wave (26, 27). In our cross sectional study, 12 patients were diagnosed with this condition. Fortunately, the majority of them have recovered, with systemic inflammation subsiding and multi-organ abnormalities relieved.

## Limitations

This study has several limitations that should be taken into consideration. Firstly, the retrospective nature of the study may have limited the clinical distribution of the cohort and sample size. There was no data on severity at admission, it is possible that asymptomatic or milder cases were not included in the study as they may have been treated at home without seeking medical attention. This may have resulted in an underrepresentation of such cases in the study cohort. Secondly, the study was conducted in southern China, and the characteristics of the setting may not be wholly representative of Chinese patients as a whole. The findings may not be generalizable to other regions or populations. Furthermore, specific vaccination coverage data for children were not available for this study. As a result, the impact of influenza and COVID-19 vaccinations on hospitalized children could not be evaluated.

This study was limited by variations in case ascertainment through positive test results due to the use of different platforms for detecting Flu A viruses, the sensitivity of antibody detection was lower than that of PCR assay.

## Conclusion

Influenza A infections have notably surged in children, coinciding with the relaxation of COVID-19 related social restrictions. During the pandemic periods of SARS-CoV-2 Omicron and Flu A virus infection, different clinical and laboratory characteristics were observed in hospitalized children, our findings may inform the prompt identification and treatment of children with a respiratory viral infection in health care facilities.

## Data availability statement

The original contributions presented in this study are included in this article/supplementary material, further inquiries can be directed to the corresponding author.

## Ethics statement

The studies involving humans were approved by the Institute Ethics Committee of Chaozhou Central Hospital (No. 2023009) and Yangjiang People’s Hospital (No. 20230003). The studies were conducted in accordance with the local legislation and institutional requirements. The Ethics Committee/Institutional Review Board waived the requirement of written informed consent for participation from the participants or the participants’ legal guardians/next of kin.

## Author contributions

FL: Writing—original draft. M-TC: Writing—review and editing. LZ: Writing—review and editing. MW: Writing—review and editing. HX: Writing—review and editing. Z-XG: Writing—review and editing. ZY: Writing—review and editing. Z-XC: Writing—review and editing. J-ZW: Writing—review and editing. L-YY: Writing—original draft.
